# Supplementary material for: Content-rich biological network constructed by mining PubMed abstracts
Source: BMC Bioinformatics. 2004 Oct 8;5:147. doi: 10.1186/1471-2105-5-147 (PMC528731; doi:10.1186/1471-2105-5-147)
Supplement: Additional File 2 — The original results of the above study (non-essential files are deleted to keep the file size under the limit set by BMC bioinformatics). [file 1471-2105-5-147-S2.bz2 › chilibotAdditionalFile2/dip50/173ID9388212E588/html/IL6ST_PTPN11.html]

 


 **IL6ST** and **PTPN11** 
  
Found 54 abstracts in PubMed, retrieved 50.  
 

 What does Google say? 
 PDF only 
| .edu only 

---

**Interactive relationship** (e.g. stimulation, inhibition, etc)

**Inhibitory relationship**- Coexpression of catalytically inactive, but not wild type, SHP 2  [ **PTPN11** ]  reduced LIFR and gp130  [ **IL6ST** ]  mediated activation of MAPK up to 75%.  Ref: 10800945 J Neurochem, 2000
- The data further suggest that SHP 2  [ **PTPN11** ]  functions normally to attenuate gp130  [ **IL6ST** ]  mediated signaling in hepatic and, perhaps, other cells by moderating JAK action.  Ref: 9488469 Mol Cell Biol, 1998
- Furthermore, we show that tyrosine 759 in gp130  [ **IL6ST** ]  is essential for both SHP2  [ **PTPN11** ]  and SOCS3 but not for SOCS1 to exert their inhibitory activities on interleukin 6 signal transduction.  Ref: 10777583 J Biol Chem, 2000
- Interestingly, one SHP2  [ **PTPN11** ]  recruiting phosphotyrosine motif in a single chain of the gp130  [ **IL6ST** ]  dimer is sufficient to mediate SHP2  [ **PTPN11** ]  association to the gp130  [ **IL6ST** ]  receptor subunit and its tyrosine phosphorylation as well as to attenuate IL 6 dependent gene induction.  Ref: 10946280 J Immunol, 2000
- A mutation of tyrosine 759, the SHP 2  [ **PTPN11** ]  binding site of gp130  [ **IL6ST** ] , abrogated the interactions of Gab1 with SHP 2  [ **PTPN11** ]  and PI 3 kinase as well as ERK2 activation.  Ref: 9632795 Mol Cell Biol, 1998
- Notwithstanding these similarities in the patterns of signaling responses elicited, mutation of the SHP 2  [ **PTPN11** ]  interaction site in G gp130  [ **IL6ST** ]  Y2F abrogated ligand activated receptor recruitment of SHP 2  [ **PTPN11** ]  as expected.  Ref: 9488469 Mol Cell Biol, 1998
- SHP2  [ **PTPN11** ]  and SOCS3 contribute to Tyr 759 dependent attenuation of interleukin 6 signaling through gp130  [ **IL6ST** ] .  Ref: 12403768 J Biol Chem, 2003
- Tyrosine 759 of gp130  [ **IL6ST** ]  is required for the negative regulation of LIF mediated signaling by both the protein tyrosine phosphatase SHP 2  [ **PTPN11** ]  and the suppressor of cytokine signaling 3 SOCS 3.  Ref: 12425940 Brain Res Mol Brain Res, 2002
- Mitogenic hyperresponsiveness of synovial cells to the LIF IL 6 family of cyto kines was caused by sustained gp130  [ **IL6ST** ]  mediated SHP 2  [ **PTPN11** ]  ras Erk activation due to impaired STAT mediated induction of suppressor of cytokine signaling SOCS proteins which normally limits gp130  [ **IL6ST** ]  signaling.  Ref: 11457894 J Exp Med, 2001
- The receptor subunit gp130  [ **IL6ST** ]  transduces multiple cell type specific activities of the leukemia inhibitory factor LIF interleukin IL 6 family of cytokines through the signal transducer and activator of transcription STAT and src homology 2 domain bearing protein tyrosine phosphatase SHP 2  [ **PTPN11** ]  ras Erk pathways.  Ref: 11457894 J Exp Med, 2001
**Stimulatory relationship**- This indicates that STAT signaling is necessary for LIF and CNTF stimulated VIP and ChAT expression and Y759 of gp130  [ **IL6ST** ]  mediates the activities of SHP 2  [ **PTPN11** ]  and SOCS 3, which act to negatively regulate STAT activity.  Ref: 12425940 Brain Res Mol Brain Res, 2002
- On IL 6 stimulation, SHP 2  [ **PTPN11** ]  and Gab1 were recruited to the gp130  [ **IL6ST** ]  subunit of the IL 6 receptor and tyrosine phosphorylated, allowing downstream signaling to the MAPK and PI3K pathways.  Ref: 11196191 Cancer Res, 2001
- Inhibition of SHP 2  [ **PTPN11** ] , either by mutating the tyrosine residue in gp130  [ **IL6ST** ]  that mediates the SHP 2  [ **PTPN11** ]  interaction, or by expression of dominant negative SHP 2  [ **PTPN11** ] , resulted in dramatic increases in gp130  [ **IL6ST** ]  dependent gene expression, through the VIP CyRE and more specifically through multimerized STAT binding sites.  Ref: 9285712 Curr Biol, 1997
- SHP 2  [ **PTPN11** ]  has more recently been shown to be tyrosine phosphorylated and recruited to the gp130  [ **IL6ST** ]  component of the ciliary neurotrophic factor receptor complex upon stimulation with CNTF.  Ref: 9497348 J Biol Chem, 1998
- Stimulation of the c fos, c jun, and egr 1 genes is essentially absent in cells expressing gp130  [ **IL6ST** ]  with a Y759F mutation, which is unable to recruit SHP 2  [ **PTPN11** ] .  Ref: 10409724 Mol Cell Biol, 1999
**Neutral relationship**- Suppressor of cytokine signaling 3 preferentially binds to the SHP 2  [ **PTPN11** ]  binding site on the shared cytokine receptor subunit gp130  [ **IL6ST** ] .  Ref: 10829066 Proc Natl Acad Sci U S A, 2000
- Protein tyrosine phosphatase 2 SHP 2  [ **PTPN11** ]  moderates signaling by gp130  [ **IL6ST** ]  but is not required for the induction of acute phase plasma protein genes in hepatic cells.  Ref: 9488469 Mol Cell Biol, 1998
- Here we demonstrate that no further tyrosines in the cytoplasmic part of gp130  [ **IL6ST** ]  are required for the phosphorylation of SHP2  [ **PTPN11** ] .  Ref: 10946280 J Immunol, 2000
- Reciprocal regulation of gastrointestinal homeostasis by SHP2  [ **PTPN11** ]  and STAT mediated trefoil gene activation in gp130  [ **IL6ST** ]  mutant mice.  Ref: 12219085 Nat Med, 2002
- Immediately after receptor complex activation, signal transducers and activators of transcription STATs 1 and 3 and the Src homology domain containing protein tyrosine phosphatase 2 SHP2  [ **PTPN11** ]  are recruited to gp130  [ **IL6ST** ]  and subsequently tyrosine phosphorylated.  Ref: 11737194 Eur J Biochem, 2001
- We show that receptor and membrane targeted SHP2  [ **PTPN11** ]  counteracts IL 6 signaling independent of SOCS3 binding to gp130  [ **IL6ST** ] .  Ref: 12403768 J Biol Chem, 2003
- Taken together, these findings indicate that Gab1 SHP2  [ **PTPN11** ]  interaction plays a crucial role in gp130  [ **IL6ST** ]  dependent longitudinal elongation of cardiomyoctes through activation of ERK5.  Ref: 12855672 Circ Res< MedlineTA>Circ Res, 2003
- Intriguingly, recent in vivo studies indicated that the SHP 2  [ **PTPN11** ]  binding site and YXXQ mediated pathways through gp130  [ **IL6ST** ]  are not mutually exclusive but affect each other a mutation at the SHP 2  [ **PTPN11** ]  binding site prolongs STAT3 activation, and a loss of STAT activation by gp130  [ **IL6ST** ]  truncation leads to sustained SHP 2  [ **PTPN11** ]  ERK MAPK phosphorylation.  Ref: 12687404 Rev Physiol Biochem Pharmacol< MedlineTA>Rev Physiol Biochem Pharmacol, 2003
- gp130  [ **IL6ST** ]  F759 F759 knockin mice carry a point mutation at the SHP2  [ **PTPN11** ]  binding site of gp130  [ **IL6ST** ]  due to the replacement of tyrosine 759 Y759 for human gp130  [ **IL6ST** ]  with phenylalanine F.  Ref: 12070777 Genes Immun, 2002
- These data suggest, that there are two, largely distinct modes of negative regulation of gp130  [ **IL6ST** ]  activity, despite the fact that both SOCS3 and SHP2  [ **PTPN11** ]  are recruited to the same site within gp130  [ **IL6ST** ] .  Ref: 12403768 J Biol Chem, 2003
- We generated a mouse line in which the src homology 2 domain bearing protein tyrosine phosphatase SHP 2  [ **PTPN11** ]  binding site of gp130  [ **IL6ST** ] , tyrosine 759, was mutated to phenylalanine gp130  [ **IL6ST** ]  F759 F759.  Ref: 12370259 J Exp Med, 2002
- Therefore, the joint pathology in gp130  [ **IL6ST** ]  DeltaSTAT mice is likely to arise from the disturbance of the otherwise balanced activation of the SHP 2  [ **PTPN11** ]  ras Erk and STAT signaling cascades emanating from gp130  [ **IL6ST** ] .  Ref: 11457894 J Exp Med, 2001
- We generated a series of knockin mouse lines, in which the cytokine receptor gp130  [ **IL6ST** ]  dependent STAT3 and or SHP2  [ **PTPN11** ]  signals were disrupted, by replacing the mouse gp130  [ **IL6ST** ]  gene with human gp130  [ **IL6ST** ]  mutant cDNAs.  Ref: 10661409 Immunity, 2000
- The gp130F759 F759 mice showed prolonged gp130  [ **IL6ST** ]  induced STAT3 activation, indicating a negative regulatory role for SHP2  [ **PTPN11** ] .  Ref: 10661409 Immunity, 2000
- Activation of gp130  [ **IL6ST** ]  Transduces Hypertrophic Signal Through Interaction of Scaffolding Docking Protein Gab1 With Tyrosine Phosphatase SHP2  [ **PTPN11** ]  in Cardiomyocytes.  Ref: 12855672 Circ Res< MedlineTA>Circ Res, 2000
- Hematopoietic growth control by gp130  [ **IL6ST** ]  is critically dependent on activation of both STAT3 and protein tyrosine phosphatase 2 SHP 2  [ **PTPN11** ] .  Ref: 9488469 Mol Cell Biol, 1998
- Signalling molecules activated downstream of gp130  [ **IL6ST** ]  in ES cells include STAT3, the protein tyrosine phosphatase SHP 2  [ **PTPN11** ] , and the mitogen activated protein kinases, ERK1 and ERK2.  Ref: 10364425 Dev Biol, 1999
- These data suggest that desensitization of vascular cells to OnM is mediated in trans and involves Tyr residue 759 in gp130  [ **IL6ST** ]  but is not mediated by either SOCS 3 or SHP 2  [ **PTPN11** ] , the only two proteins currently known to bind to gp130  [ **IL6ST** ]  at this site.  Ref: 12724316 J Biol Chem< MedlineTA>J Biol Chem, 1999
- gp130  [ **IL6ST** ]  and LIFR contain consensus binding motifs for the protein tyrosine phosphatase SHP 2  [ **PTPN11** ]  surrounding tyrosines 118 and 115 Y118 and Y115 of their cytoplasmic domains, respectively.  Ref: 10800945 J Neurochem, 2000
- Tyrosine 759 of gp130  [ **IL6ST** ] , the signal transducing subunit of the IL 6R complex, is essential for the phosphorylation of SHP2  [ **PTPN11** ] .  Ref: 10946280 J Immunol, 2000
- Mutational analysis of the cytoplasmic domain of gp130  [ **IL6ST** ]  revealed that the tyrosine residue of gp130  [ **IL6ST** ]  responsible for STAT3 activation is necessary for self renewal of ES cells, while that required for SHP2  [ **PTPN11** ]  and MAP kinase activation was dispensable.  Ref: 10428964 EMBO J, 1999
- The only high affinity ligand found corresponded to the region of gp130  [ **IL6ST** ]  centered around phosphotyrosine 757 pY757, previously shown to be a docking site for the tyrosine phosphatase SHP 2  [ **PTPN11** ] .  Ref: 10829066 Proc Natl Acad Sci U S A, 2000
- We conclude that normal Shp 2  [ **PTPN11** ]  function is critical for the initial step of ES cell differentiation to mesoderm and to hemangioblasts and acts within the LIF gp130  [ **IL6ST** ]  Stat3 pathway to maintain a proper balance of ES cell differentiation, pluripotency, and apoptosis.  Ref: 12791646 Blood< MedlineTA>Blood, 2000
- Ciliary neurotrophic factor, along with other neuropoietic cytokines, signals through the shared receptor subunit gp130  [ **IL6ST** ]  1 3, leading to the tyrosine phosphorylation of a number of substrates, including the transcription factors STAT1 and STAT3 and the protein tyrosine phosphatase SHP 2  [ **PTPN11** ]  8.  Ref: 9285712 Curr Biol, 1997
- Ligand binding to the receptor complex leads to tyrosine phosphorylation and activation of Janus kinases Jak, phosphorylation of the signal transducing subunit gp130  [ **IL6ST** ] , followed by recruitment and phosphorylation of the signal transducer and activator of transcription factors STAT3 and STAT1 and the src homology domain SH2 containing protein tyrosine phosphatase SHP2  [ **PTPN11** ] .  Ref: 9794795 Biochem J, 1998
- JAK tyrosine kinase is a key molecule that can initiate multiple signal transduction pathways by inducing the tyrosine phosphorylation of the cytokine receptor, gp130  [ **IL6ST** ]  in the case of IL 6, on which several signaling molecules are recruited, including STAT, a signal transducer and activator of transcription, and SHP 2  [ **PTPN11** ] , which links to the Ras MAP kinase pathway.  Ref: 9505191 Int Rev Immunol, 1998

**Non-interactive relationship** (e.g. studied together, co-existance, homology, etc.)

- Here we present data on the dose dependence, kinetics and kinase requirements for SHP2  [ **PTPN11** ]  phosphorylation after the activation of the signal transducer, gp130  [ **IL6ST** ] , of the IL 6 type family receptor complex.  Ref: 9794795 Biochem J, 1998
- We also tested whether the tyrosine 759 motifs in both subunits of the gp130  [ **IL6ST** ]  dimer are required for SHP2  [ **PTPN11** ]  association and tyrosine phosphorylation.  Ref: 10946280 J Immunol, 2000
- ... the Y759 SHP 2  [ **PTPN11** ]  mediated cascade by gp130  [ **IL6ST** ]  stimulation has growth enhancing effects.  Ref: 12687404 Rev Physiol Biochem Pharmacol< MedlineTA>Rev Physiol Biochem Pharmacol, 2000
- Overexpression of the enzymatically inactive SHP 2  [ **PTPN11** ]  enhanced the signaling by the wild type but not by the Y2F mutant G gp130  [ **IL6ST** ]  receptor.  Ref: 9488469 Mol Cell Biol, 1998
- The Csk effect is attributed to prevention of Src kinases from phosphorylating gp130  [ **IL6ST** ]  at the docking site for the signal moderating protein tyrosine phosphatase SHP 2  [ **PTPN11** ] .  Ref: 10462375 Hepatology, 1999
- These data suggest that SHP 2  [ **PTPN11** ]  has a negative role in gp130  [ **IL6ST** ]  signaling by modulating STAT mediated transcriptional activation.  Ref: 9285712 Curr Biol, 1997
- M Ernst and colleagues generated and studied knock in mice gp130DeltaSTAT DeltaSTAT, in which all STAT binding sites sites binding signal transducers and activators of transcription were deleted from their gene encoding gp130  [ **IL6ST** ]  but binding sites for both Janus kinases JAKs and for the protein tyrosine phosphatase 2 SHP 2  [ **PTPN11** ]  were preserved.  Ref: 12010564 Arthritis Res, 2002
- Additionally, LIF enhances association of SHP 2  [ **PTPN11** ]  with the gp130  [ **IL6ST** ]  subunit of the LIF receptor signaling complex.  Ref: 9507023 J Biol Chem, 1998
- To characterize the role of SHP 2  [ **PTPN11** ]  in the regulation of gp130  [ **IL6ST** ]  stimulated gene expression, we examined the regulation of the VIP CyRE in two systems that prevented ligand dependent SHP 2  [ **PTPN11** ]  phosphorylation.  Ref: 9285712 Curr Biol, 1997
- Activation of the protein tyrosine phosphatase SHP2  [ **PTPN11** ]  via the interleukin 6 signal transducing receptor protein gp130  [ **IL6ST** ]  requires tyrosine kinase Jak1 and limits acute phase protein expression.  Ref: 9794795 Biochem J, 1998
- The key components involved are the signal transducing receptor subunit gp130  [ **IL6ST** ] , the Janus kinases Jak1, Jak2 and Tyk2, STAT1 and STAT3 of the family of signal transducers and activators of transcription, the protein tyrosine phosphatase SHP2  [ **PTPN11** ]  and the suppressors of cytokine signalling SOCS1, SOCS2 and SOCS3.  Ref: 10491180 Eur J Biochem, 1999
- In this respect murine fibroblasts lacking exon 3 of SHP2  [ **PTPN11** ]  are not sensitive to TNF alpha, indicating that functional SHP2  [ **PTPN11** ]  and its recruitment to gp130  [ **IL6ST** ]  are key events in inhibition of IL 6 dependent STAT activation by TNF alpha.  Ref: 12817006 J Immunol, 2003
- Furthermore, activation of p38 mitogen activated protein kinase is shown to be essential for the inhibitory effect of TNF alpha on IL 6 signaling and TNF alpha dependent recruitment of SHP2  [ **PTPN11** ]  to gp130  [ **IL6ST** ] .  Ref: 12817006 J Immunol, 2003
- This study shows that in hepatoma cells, the recruitment and tyrosine phosphorylation of SHP 2  [ **PTPN11** ] , but not SHC, is the primary signaling event associated with the activation of MAP kinases by gp130  [ **IL6ST** ] .  Ref: 10409724 Mol Cell Biol, 1999
- However, the extent to which hematopoiesis is dependent upon activation of a particular gp130  [ **IL6ST** ]  signaling pathway, namely STAT1 3 or SHP2  [ **PTPN11** ]  MAPK, is unknown.  Ref: 12423677 Exp Hematol, 2002
- Interestingly, gp130  [ **IL6ST** ]  associated SHP 2  [ **PTPN11** ]  phosphorylation was detected in the IL 6 induced CESS cells but not myeloma cell lines.  Ref: 12040451 Leukemia, 2002
- Synovial cells from this mouse exhibited mitogenic hyper responsiveness to cytokines of the LIF IL 6 family, a phenomenon that was caused by sustained gp130  [ **IL6ST** ]  mediated SHP 2  [ **PTPN11** ]  Ras Erk activation due to a defect in the induction of SOCS 1 suppressor of cytokine signaling 1.  Ref: 12010564 Arthritis Res, 2002
- Dissection of signaling cascades through gp130  [ **IL6ST** ]  in vivo reciprocal roles for STAT3 and SHP2  [ **PTPN11** ]  mediated signals in immune responses.  Ref: 10661409 Immunity, 2000
- Prior activation of mitogen activated protein kinases by phorbol 13 myristate 12 acetate PMA results in an inhibition of interleukin IL 6 induced activation of the Janus kinase signal transducer and activator of transcription STAT signaling pathway which is most likely mediated by the induction of suppressor of cytokine signaling 3 and requires the specific SHP2  [ **PTPN11** ]  binding site Y759 of the IL 6 signal transducer gp130  [ **IL6ST** ] .  Ref: 10922477 FEBS Lett, 2000
- On the other hand, SOCS3 inhibits signaling in cells expressing a truncated SHP2  [ **PTPN11** ]  protein, which is not recruited to gp130  [ **IL6ST** ] .  Ref: 12403768 J Biol Chem, 2003
- In this study we demonstrate that stimulation of macrophages and fibroblast cell lines with TNF alpha causes the recruitment of SHP2  [ **PTPN11** ]  to the gp130  [ **IL6ST** ]  signal transducing subunit and leads to tyrosine phosphorylation of SHP2  [ **PTPN11** ]  and gp130  [ **IL6ST** ] .  Ref: 12817006 J Immunol, 2003
- Negative regulation of gp130  [ **IL6ST** ]  signalling mediated through tyrosine 757 is not dependent on the recruitment of SHP2  [ **PTPN11** ] .  Ref: 12593670 Biochem J, 2003
- This phosphorylation depends on Tyr 759 in the cytoplasmatic domain of gp130  [ **IL6ST** ] , since a Tyr 759 >Phe exchange abrogates SHP2  [ **PTPN11** ]  activation and in turn leads to elevated and prolonged STAT3 and STAT1 activation as well as enhanced acute phase protein gene induction.  Ref: 9794795 Biochem J, 1998
- SOCS3 exerts its inhibitory function on interleukin 6 signal transduction through the SHP2  [ **PTPN11** ]  recruitment site of gp130  [ **IL6ST** ] .  Ref: 10777583 J Biol Chem, 2000
- Here, we evaluated the functions of the two major signaling pathways, the signal transducers and activators of transcription 1 and 3 STAT1 3 and the Src homology tyrosine phosphatase 2 SHP2  [ **PTPN11** ]  Ras ERK, emanating from the common signal transducer, gp130  [ **IL6ST** ] , in the gastrointestinal tract.  Ref: 12219085 Nat Med, 2002
- Functional analysis of gp130  [ **IL6ST** ]  in rat hepatoma cells by using transduced chimeric G CSFR gp130  [ **IL6ST** ]  receptor constructs demonstrates that SHP 2  [ **PTPN11** ] , the Src homology 2 domain containing protein tyrosine phosphatase, acts as a negative regulator of the JAK STAT signaling in part by downregulating JAK activity, thereby indirectly moderating the induction of STAT3 dependent APP genes.  Ref: 10409724 Mol Cell Biol, 1999
- Early intracellular events stimulated by the sIL 11R including phosphorylation of gp130  [ **IL6ST** ] , STAT 3, and SHP 2  [ **PTPN11** ]  were similar to signaling through the transmembrane IL 11R.  Ref: 9373251 Blood, 1997
- These results indicate that gp130  [ **IL6ST** ]  signaling for APP gene induction in hepatic cells differs qualitatively from that controlling the proliferative response in hematopoietic cells in not being strictly dependent on SHP 2  [ **PTPN11** ] .  Ref: 9488469 Mol Cell Biol, 1998
- Gp130  [ **IL6ST** ]  757F mice, with a knock in mutation abrogating SHP2  [ **PTPN11** ]  Ras ERK signaling, developed gastric adenomas by three months of age.  Ref: 12219085 Nat Med, 2002
- Stimulation of the IL 6R complex leads to Src homology domain containing tyrosine phosphatase 2 SHP2  [ **PTPN11** ]  recruitment to the receptor subunit gp130  [ **IL6ST** ]  and its subsequent tyrosine phosphorylation.  Ref: 10946280 J Immunol, 2000
- Since recruitment of the tyrosine phosphatase Src homology protein tyrosine phosphatase 2 SHP2  [ **PTPN11** ]  to the signal transducing receptor subunit gp130  [ **IL6ST** ]  attenuates IL 6 mediated STAT activation, we were interested in whether TNF alpha also induces the association of SHP2  [ **PTPN11** ]  to the gp130  [ **IL6ST** ]  receptor subunit.  Ref: 12817006 J Immunol, 2003
- A receptor carrying tyrosine module Y759 of gp130  [ **IL6ST** ]  effectively mediated activation of the phosphatase SHP 2  [ **PTPN11** ]  but did not alter cell growth.  Ref: 10391682 Oncogene, 1999
- Moreover, disengagement of SHP 2  [ **PTPN11** ]  from gp130  [ **IL6ST** ]  signaling not only enhances APP gene induction but also further reduces cell proliferation, in part correlated with the attenuated expression of immediate early response genes.  Ref: 10409724 Mol Cell Biol, 1999
- A chimaeric receptor in which tyrosine 118 in the gp130  [ **IL6ST** ]  cytoplasmic domain was mutated did not engage SHP 2  [ **PTPN11** ]  and failed to activate ERKs.  Ref: 10364425 Dev Biol, 1999
- Suppressor of cytokine signaling 3 SOCS 3 and the protein tyrosine phosphatase SHP 2  [ **PTPN11** ]  both regulate signaling by cytokines of the interleukin 6 family, and this is dependent upon recruitment to tyrosine 757 in the shared cytokine receptor subunit gp130  [ **IL6ST** ] .  Ref: 12119038 Biochemistry, 2002
- Cells transfected with a chimaeric receptor containing the SHP2  [ **PTPN11** ]  selective gp130  [ **IL6ST** ]  intracellular domain showed an enhanced response to cytokine stimulation, which was similar to that shown by a chimaeric gp130  [ **IL6ST** ]  receptor mutant carrying a Y757F point mutation that failed to recruit either SOCS3 or SHP2  [ **PTPN11** ] .  Ref: 12593670 Biochem J, 2003
- To investigate whether induction of APP genes has a similar requirement for SHP 2  [ **PTPN11** ] , we constructed two chimeric receptors, G gp130  [ **IL6ST** ]  and G gp130  [ **IL6ST** ]  Y2F, consisting of the transmembrane and cytoplasmic domains of gp130  [ **IL6ST** ]  harboring either a wild type or a mutated SHP 2  [ **PTPN11** ]  binding site, respectively, fused to the extracellular domain of the granulocyte colony stimulating factor G CSF receptor.  Ref: 9488469 Mol Cell Biol, 1998
- In the present study we demonstrate that in addition to STAT3, also tyrosine phosphorylation of STAT1, signal transducer gp130  [ **IL6ST** ] , and phosphotyrosine phosphatase SHP2  [ **PTPN11** ]  underlies negative regulation by MAP kinases.  Ref: 10764798 J Biol Chem, 2000
- In the present study, we show the design of a mutant gp130  [ **IL6ST** ]  receptor that can recruit SHP2  [ **PTPN11** ] , but not SOCS3.  Ref: 12593670 Biochem J, 2003
- To determine whether duration is functionally significant, we transduced EC with a chimeric receptor containing extracellular domains of platelet derived growth factor receptor beta and intracellular regions of gp130  [ **IL6ST** ] , the signaling subunit of the OnM receptor, mutated to prevent binding of the tyrosine phosphatase SHP 2  [ **PTPN11** ] .  Ref: 11777927 J Biol Chem, 2002
- The subsequent recruitment of the common signal transducer gp130  [ **IL6ST** ]  and activation of the JAK STAT and SHP 2  [ **PTPN11** ]  Ras mitogen activated protein kinase MAPK pathways are responsible for the majority of cellular responses elicited by IL 6 cytokines.  Ref: 12764151 J Biol Chem< MedlineTA>J Biol Chem, 2002
- We previously found that the adapter protein Gab1 110 kD is tyrosine phosphorylated and forms a complex with SHP 2  [ **PTPN11** ]  and PI 3 kinase upon stimulation through either the interleukin 3 receptor IL 3R or gp130  [ **IL6ST** ] , the common receptor subunit of IL 6 family cytokines.  Ref: 10068651 Blood, 1999
- In this context the cytoplasmic SHP2  [ **PTPN11** ]  SOCS3 recruitment site of gp130  [ **IL6ST** ]  tyrosine 759 is shown to be important for the inhibitory effects of TNF alpha, since mutation of this residue completely restores IL 6 stimulated activation of STAT3 and, consequently, of a STAT3 dependent promoter.  Ref: 12817006 J Immunol, 2003
- Besides SHP2  [ **PTPN11** ] , SOCS3 also interacts with the Tyr P 759 peptide of gp130  [ **IL6ST** ] .  Ref: 10777583 J Biol Chem, 2000
- These results demonstrate that the recruitment of SHP2  [ **PTPN11** ]  alone is not sufficient for Y757 dependent negative regulation of gp130  [ **IL6ST** ]  signalling.  Ref: 12593670 Biochem J, 2003
